# Clinical use of 3D printed models for anterior communicating artery aneurysm clipping: a prospective cohort study

**DOI:** 10.3389/fsurg.2025.1639912

**Published:** 2025-12-03

**Authors:** Chudi Feng, Jianli Wang, Keqiong Lv, Changming Dong, Jinquan Li, Li Zhao, Ying Duan, Honghong Shao, Zigang Yuan

**Affiliations:** 1 The First Affiliated Hospital of Shaoxing University, Shaoxing, Zhejiang, China; 2 The Department of Neurosurgery, Shaoxing People’s Hospital, Shaoxing, Zhejiang, China; 3 The Department of Radiology, Shaoxing People’s Hospital, Shaoxing, Zhejiang, China

**Keywords:** anterior communicating artery aneurysm, 3D printing, aneurysm clipping surgery, surgical simulation, complex aneurysm model

## Abstract

**Objective:**

The complex anatomy of anterior communicating artery aneurysms (ACoA) makes microsurgical clipping challenging. This study assessed the clinical value of 3D printed models based on digital subtraction angiography (DSA) in the surgical management of ACoA aneurysms, with a comprehensive analysis of ruptured and unruptured cases.

**Methods:**

A prospective cohort study was conducted from 2022 to 2023, involving 60 patients with ACoA aneurysms. The study included both ruptured (*n* = 42) and unruptured (*n* = 18) aneurysms. Patients were divided into two groups: a control group (*n* = 30) using traditional 2D DSA imaging and 3D rotational angiography displays and a model group (*n* = 30) utilizing 3D printed models. Patient characteristics, including comorbidities such as hypertension, smoking status, and diabetes, as well as Hunt and Hess scores for ruptured cases, were recorded. For ruptured cases, Fisher grades, Hunt and Hess scores, and presence of hydrocephalus were documented. Primary outcomes included residual aneurysm neck, parent artery stenosis, and modified Rankin Scale (mRS) at 14 days post-surgery. Secondary outcomes encompassed intraoperative complications, diagnostic accuracy, operative duration, and perioperative clinical parameters. Temporary clip usage and duration were recorded, and intraoperative vessel patency was verified using Doppler ultrasonography and indocyanine green video angiography.

**Results:**

At 14 days postoperatively, the model group demonstrated significantly lower rates of residual aneurysm neck (0% vs. 20%, *p* = 0.012) and parent artery stenosis (3.33% vs. 23.33%, *p* = 0.026). Fewer patients in the model group had mRS ≥ 3 (10% vs. 33.33%, *p* = 0.028). Intraoperative complications were reduced in the 3D model group (6.67% vs. 26.67%, *p* = 0.038), with significantly shorter operation duration (264.47 ± 52.27 vs. 313.10 ± 59.90 min, *p* = 0.001). The model group had higher preoperative diagnostic accuracy (93.33% vs. 70%, *p* = 0.02) With the aid of 3D models, surgical precision and outcomes are improved.

**Conclusion:**

3D printed models derived from DSA imaging significantly enhance the surgical management of ACoA aneurysms, offering improved diagnostic accuracy, reduced complications, and better functional outcomes. The average production time of 2–8 h and cost of approximately $120 USD per model make this approach feasible even for time-sensitive cases. These findings highlight the potential of patient-specific 3D models as a valuable adjunct in the management of complex ACoA aneurysms.

## 1 Introduction

Anterior communicating artery (ACoA) aneurysms are the most common type of intracranial aneurysms, accounting for approximately 25%–38% of all cases, and due to the complex vascular anatomical structure of the ACoA complex, ACoA aneurysms are considered to be among the most challenging situations among all aneurysms (1, 2). Microsurgical clipping is an important treatment modality for ACoA aneurysms, for example, ACoA aneurysms projecting towards the optic nerve are preferably treated with microsurgical clipping (3), and clipping may also be the best treatment option for patients with recurrent ACoA aneurysms after endovascular treatment (4). Compared to endovascular treatments such as coil embolization and stent-assisted coiling, microsurgical clipping offers more robust treatment outcomes with lower rates of recurrence and re-treatment risk, however unfortunately, it carries a higher risk of complications (5, 6). Therefore, enhancing the surgical skills of neurosurgeons and minimizing the risk of operative complications is a key issue that needs to be addressed.

For neurosurgeons, especially those in training, integrating theoretical knowledge with surgical skills poses certain challenges (7). Image-based surgical training is often abstract and difficult to grasp. Traditionally, preoperative planning for ACoA aneurysm surgery relies on the interpretation of two-dimensional (2D) digital subtraction angiography (DSA) images and three-dimensional (3D) rotational angiography. Although these modalities offer valuable spatial information, they still fall short in conveying the complex three-dimensional anatomical relationships that are critical for surgical decision-making. While 3D rotational angiography provides superior visualization compared to 2D imaging, surgeons are still required to mentally reconstruct these images during procedures, lacking the tangible spatial cues afforded by physical models. In this context, 3D printing technology serves as an effective modeling tool. 3D printing can create patient-specific vascular models to scale based on imaging data such as computed tomography angiography (CTA) and DSA, accurately replicating the shape, size, orientation, and supplying arteries of an aneurysm (8, 9). Such models facilitate detailed preoperative planning, support the design of optimal surgical approaches, aid in selecting appropriate clips, and help anticipate potential intraoperative risks and challenges (10, 11). Ultimately, they provide an effective means to bridge theoretical knowledge and practical surgical skills.

Several aspects distinguish our study from previous investigations in this field. First, we specifically focus on ACoA aneurysms, which present unique anatomical and surgical challenges compared to other intracranial aneurysms. Second, we employ DSA as the source imaging modality rather than CTA (12, 13). DSA can provide 3D images with sub-millimeter spatial resolution, offering better, resolution for the identification of small perforating arteries and distant fine branches than CTA (14), thus avoiding under-continuity or fractures in 3D-printed models. In addition, DSA can capture hemodynamics. When vascular lesions are perfused by two or more vessels, imaging of a single vessel cannot provide a complete anatomical image. The fusion of DSA images of the two feeding arteries can well restore the blood supply of ACoA aneurysms (15). Third, we comprehensively evaluate multiple clinical outcomes including residual aneurysm necks, parent artery stenosis, and functional status, providing a more robust assessment of clinical utility than previous studies that primarily focused on surgical planning and training benefits.

This study aims to clarify the application value of 3D printed models generated from DSA fusion images of bilateral internal carotid arteries in assisting the clipping of ACoA aneurysms, and how they may effectively enhance the precision and safety of the surgery in clinical practice. By analyzing a range of perioperative and postoperative outcomes, we seek to provide comprehensive evidence for the integration of this technology into standard neurosurgical practice for complex ACoA aneurysms.

## 2 Patients and methods

### 2.1 Patient enrollment

This single-center prospective cohort observational study was approved by the Ethics Committee of Shaoxing People's Hospital (NO.2021-K-Y348-01). The study involved 60 patients with ACoA aneurysms (42 ruptured, 18 unruptured) treated at the hospital's Department of Neurosurgery between 2022 and 2023.

The inclusion criteria for the study were as follows: (1) age ≥ 18, (2) Diagnosed with ACoA aneurysm, (3) Preoperative DSA imaging performed, (4) Undergo aneurysm clipping surgery. Exclusion criteria included: (1) Combined with other intracranial lesions, such as intracranial tumors, cerebral arteriovenous malformations, etc., (2) History of previous craniotomy, (3) History of endovascular treatment, (4) Recurrent aneurysms.

Patient allocation to study groups was prospectively determined based on consecutive enrollment and patient consent. Patients were offered the option of using 3D printed models as a preoperative planning tool after thorough explanation of the technology, its potential benefits, and associated costs. Those who consented formed the model group, while those who declined or preferred standard care without 3D models formed the control group. To minimize selection bias, we included controls in the unmodeled group using sequential propensity score matching based on age, aneurysm size, rupture status, and Hunt and Hess grade (for ruptured cases). Pairs were matched using 1: 1 nearest neighbor matching with a caliper width equal to 0.2 of the standard deviation of the logit of the propensity score. When the number of patients in each group reached 30, Patient enrollment was discontinued. All surgeries were performed by the same experienced neurosurgeon (with over 15 years of experience in cerebrovascular surgery) to minimize operator variability.

For ruptured aneurysms, we documented the Fisher grade (distribution: grade 1: 5 patients, grade 2: 9 patients, grade 3: 15 patients, grade 4: 13 patients), Hunt and Hess scale, and relevant comorbidities (hypertension: 58.3%, diabetes mellitus: 23.3%, smoking history: 38.3%). The presence of hydrocephalus was noted in 19 patients with ruptured aneurysms (45.2%). For all cases, we recorded detailed aneurysm characteristics including size, neck width, dome-to-neck ratio, projection direction (anterior, posterior, superior, inferior, or complex), and relationship to surrounding vasculature.

Table 1 presents comprehensive patient demographics, revealing no significant differences in age, sex, aneurysm characteristics, comorbidities, Fisher grades (for ruptured cases), Hunt and Hess grades, or preoperative modified Rankin Scale (mRS) scores between the groups (*p* > 0.05 for all comparisons).

**Table 1:** Baseline characteristics of the patients.

Characteristic	Control group (*n* = 30)	Model group (*n* = 30)	*P*
Demographics			
Age	58.63 ± 9.50	58.27 ± 10.88	0.890
Gender			0.793
Male	12 (40%)	13 (43.3%)	
Female	18 (60%)	17 (56.7%)	
Aneurysm status			1.000
Ruptured	21 (70%)	21 (70%)	
Unruptured	9 (30%)	9 (30%)	
Fisher grade (ruptured cases)			0.918
Grade 1	3 (14.3%)	2 (9.5%)	
Grade 2	4 (19.0%)	5 (23.8%)	
Grade 3	7 (33.3%)	8 (38.1%)	
Grade 4	7 (33.3%)	6 (28.6%)	
Hunt and Hess grade (ruptured cases)			0.856
Grade 1	5 (23.8%)	4 (19.0%)	
Grade 2	7 (33.3%)	8 (38.1%)	
Grade 3	6 (28.6%)	6 (28.6%)	
Grade 4	3 (14.3%)	3 (14.3%)	
Hydrocephalus (ruptured cases)	10 (47.6%)	9 (42.9%)	0.757
Comorbidities			
Hypertension	18 (60%)	17 (56.7%)	0.793
Diabetes mellitus	8 (26.7%)	6 (20%)	0.542
Smoking history	12 (40%)	11 (36.7%)	0.791
Aneurysm characteristics			
Aneurysm neck size (mm)	3.71 ± 1.72	3.18 ± 1.26	0.18
Aneurysm maximum diameter (mm)	4.67 ± 2.48	4.04 ± 1.75	0.428
Preoperative mRS			0.436
≥3	15 (50%)	12 (40%)	
<3	15 (50%)	18 (60%)	

### 2.2 3D printed model preparation

In the control group, surgical planning was based on standard 2D DSA images and 3D rotational angiography displays viewed on workstation monitors. Surgeons analyzed these images to understand aneurysm morphology and plan the surgical approach. In the model group, we created physical 3D printed models from DSA data as follows: 3D models are printed based on the DSA imaging of bilateral internal carotid arteries. Initially, Inspace-3D/3D-Fusion software is used to analyze and correct the three-dimensional imaging of the dual vessels so that the three-dimensional images are anatomically aligned. The data is then saved in DICOM format. The saved data undergoes three-dimensional reconstruction using MIMICS software; during this process, the denser cranial bone data is used as a positioning basis to automatically match and merge the dual vessel data. The required cerebral vascular area is selected and saved in STL file format. Since the current file represents the vascular lumen shown by the contrast agent, 3dsMax2017 software is utilized to convert it into a hollow model. A 0.2 mm thick outer shell is added to the exterior of the hollow model to simulate the vascular wall, and it is exported in STL file format. To quickly create models for surgical guidance, we performed a localized extraction of the ACoA aneurysm complex and generated a new STL file. Subsequently, the STL file was input into the SLA600 3D printer (China, LianTai), using SLA-9200 high-toughness epoxy resin (China, LianTai) to print the corresponding 3D model.

The entire process, from obtaining DSA images to delivering the final model, took approximately 2–8 h. Once patient-specific DSA data is obtained, it is immediately sent to engineers. DSA image processing, 3D reconstruction and segmentation, model design, and refinement are typically completed in less than 100 min. Producing a complete cerebrovascular model via 3D printing takes approximately 6 h. Depending on complexity and urgency, a patient-specific model can be completed within 2–8 h. The average material cost per model was approximately ¥850 RMB (≈$120 USD), with additional personnel costs estimated at ¥1,000–1,400 RMB (≈$140–200 USD) per case.

### 2.3 Utilization of 3D printed models in surgical practice

Upon receiving the patient-specific 3D printed model, two neurosurgeons and two radiologists thoroughly assessed it. They diagnose the aneurysm's morphology, size, and location, and evaluate its relationship with surrounding structures, with particular attention to perforator arteries and dominant A1 segment.

The key advantage of 3D printed models over 3D rotational angiography lies in their tangible nature. Unlike viewing 3D images on a screen, physical models allow surgeons to: (1) handle and rotate the model freely to view from any angle without being constrained by preset viewing angles; (2) physically test clip placement by applying actual aneurysm clips to the model, assessing fit, angle, and potential impingement on adjacent vessels; (3) simulate the surgical approach by viewing the model from the operative perspective; and (4) better appreciate spatial relationships through tactile feedback.

**Figure 1:**
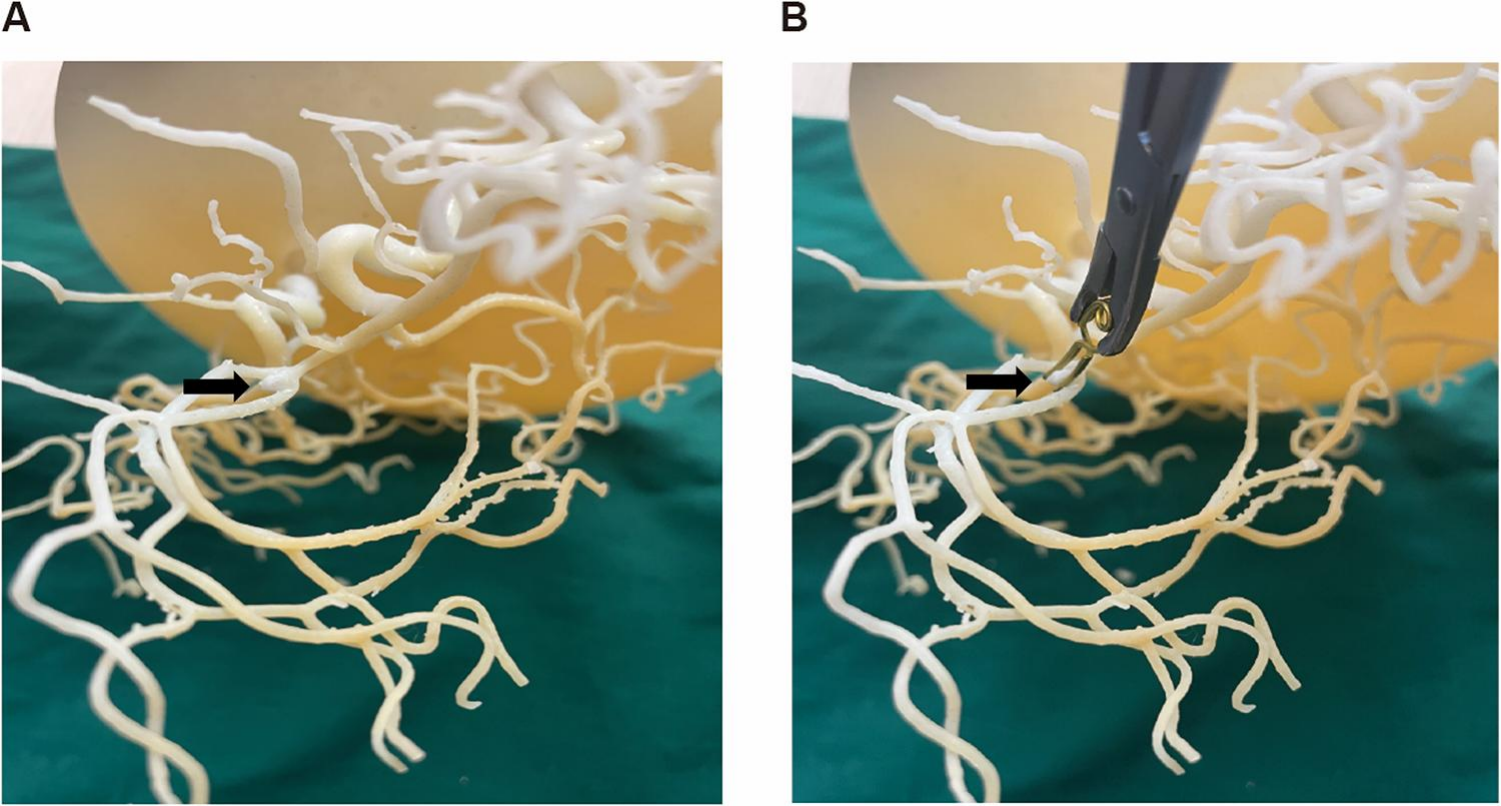
Neurosurgeon practice clipping on a patient specific 3D-printed model of the ACoA aneurysm complex. **(A)** Patient specific 3D-printed model of the ACoA aneurysm complex. **(B)** Doctors practice aneurysm clipping before surgery.

Utilizing the 3D printed model and preoperative data, the attending physician determines the surgical approach (right vs. left pterional), aneurysm clip type and size, and optimal clipping technique, while assessing potential intraoperative and postoperative challenges to formulate the surgical plan. The surgeon performs simulated clipping on the model using actual aneurysm clips of various sizes and configurations. During this simulation, the surgeon tests different clip angles and positions to achieve complete aneurysm neck obliteration while ensuring preservation of parent vessels and perforators. This hands-on trial allows identification of the optimal clip size, type (straight, curved, fenestrated), and application angle before entering the operating room (Figure 1). Subsequently, the attending physician uses the patient's 3D printed model to explain the surgical process and risks to the patient and their family, enhancing their understanding of the condition. During the surgery, the 3D printed model serves as physical reference, helping the surgeon accurately locate the aneurysm and successfully perform the aneurysm clipping procedure.

### 2.4 Surgical technique and intraoperative management

All patients underwent microsurgical clipping via pterional craniotomy with the side of approach determined based on aneurysm dome projection, dominant A1 segment, and anatomical considerations. For ruptured aneurysms with hydrocephalus, external ventricular drainage was placed prior to definitive aneurysm treatment.

Intraoperative neurophysiological monitoring was employed in all cases. During surgery, meticulous attention was given to arachnoid dissection and exposure of the aneurysm complex. Temporary clipping of parent vessels (A1 segments or ACoA) was used when necessary, with the duration meticulously recorded and limited to less than 10 min to prevent ischemic complications.

After aneurysm clipping, microvascular Doppler ultrasonography and indocyanine green (ICG) video angiography was routinely used to verify complete aneurysm obliteration and parent vessel patency. Special attention was paid to preserving perforator arteries, particularly the recurrent artery of Heubner and hypothalamic perforators. If video angiography revealed residual aneurysm filling or parent vessel compromise, clip repositioning was performed immediately.

The surgeon's handedness (right-handed in all cases in this study) was recorded along with the approach side (right-sided approach: 38 cases, 63.3%; left-sided approach: 22 cases, 36.7%) to analyze any potential correlation with surgical outcomes.

### 2.5 Postoperative management and evaluation

Postoperatively, all patients received standard care in the neurosurgical intensive care unit with continuous monitoring of vital signs and neurological status. For ruptured aneurysms, management of cerebral vasospasm included oral nimodipine (60 mg every 4 h), maintenance of euvolemia, and induced hypertension for symptomatic vasospasm.

Delayed cerebral ischemia was diagnosed based on (1) the appearance of new focal neurological signs or deterioration in level of consciousness lasting for at least 1 h, (2) exclusion of other causes of neurological deterioration through clinical assessment and neuroimaging, and (3) confirmation of vasospasm through transcranial Doppler ultrasonography or CT/DSA angiography when possible.

Postoperative CT angiography was performed within 24 h after surgery to assess immediate results and detect potential complications. Follow-up DSA was conducted at 14 days post-surgery to evaluate aneurysm occlusion status and parent vessel patency.

### 2.6 Observation content

Postoperative angiography to assess the relationship between the aneurysm and its parent artery is an important indicator for judging the quality of surgery (16). Fourteen days postoperatively, patients underwent the DSA examination to assess the presence of any residual aneurysm neck or arterial stenosis associated with the aneurysm. The DSA images were independently evaluated by two experienced neuroradiologists who were blinded to the group allocation. The Cohen's Kappa value was calculated between the two doctors to indicate the consistency of the evaluation results. If the two reviewers disagreed on a case, they would review it together to reach a consensus. If they could not reach a consensus after review, the case would be arbitrated by a third senior neuroradiologist who was blinded to the grouping information. The arbitrator's assessment would be the final decision for the case in subsequent analyses. Residual aneurysm neck was defined as any filling of the aneurysm sac or neck on follow-up angiography. Parent artery stenosis was defined as any narrowing (>20%) of the parent vessel lumen compared to the preoperative state. In addition, mRS scores at 14 days post-operation was assessed to determine surgical outcomes in both groups. This data will serve as the primary endpoint.

Detailed observations during surgery included aneurysm morphology, neck size and orientation, surrounding anatomical structures, and alignment of aneurysm clips with preoperative plans. Discrepancies between preoperative diagnosis and intraoperative findings were recorded and classified as errors in morphology (error in measurement of the maximum aneurysm diameter exceeding 10%), neck measurement (error in measurement of the aneurysm neck width exceeding 1 mm), orientation (error in orientation exceeding 25°), adjacency relationship (missing or misidentifying important perforating vessels, critical small vessels, cranial nerves, etc. adherent to the aneurysm neck/tumor body), or aneurysm clip selection (needing a replacement). These indicators will serve as secondary endpoint.

Exploratory outcomes included surgical duration of anesthesia to completion of suturing (measured in minutes), intraoperative blood loss (measured in milliliters), Length of hospital stay (measured in day), and the incidence of perioperative complications. These complications encompassed aneurysm rupture, stroke, postoperative rebleeding, epilepsy, cerebral vasospasm, and brain herniation. All outcomes were statistically analyzed to provide a comprehensive overview of procedural efficacy and safety.

### 2.7 Statistical method

All Data were analyzed using SPSS statistical software (version 25.0, IBM Corp., Armonk, NY, USA). Quantitative data were expressed as mean ± standard deviation. Normally distributed quantitative data were analyzed using the *T*-test. Cohen's d and 95% confidence interval (95% CI) were calculated to express the effect size. Non-normally distributed quantitative data were expressed as Median (quartiles) and analyzed using the Mann–Whitney test. *Z* value and 95% CI represent the effect size. Categorical data were expressed as counts (percentage) and analyzed using the Chi-square test. The odds ratio (OR) and 95% CI were calculated to express the effect size. The level of significance was set at *P* = 0.05.

## 3 Results

### 3.1 Comparison of short-term postoperative surgical effects between the two groups

The Cohen's Kappa value for the agreement between the two neuroradiologists regarding residual aneurysm neck was 0.677 (95% CI 0.373–0.961). The Cohen's Kappa value for the agreement regarding parent artery stenosis was 0.795 (95% CI 0.572–1.018). The observed agreement was good (17). All discrepant results were resolved through joint review. DSA examination was performed for 14 days postoperatively, revealing that all aneurysms were completely clipped in patients of model group. However, one patient (3.33%) presented with parent artery stenosis. In contrast, seven patients (23.33%) in the control group experienced parent artery stenosis, and six patients (20.00%) had residual aneurysm necks. The incidence of residual aneurysm necks and arterial stenosis showed a significant difference between the two groups (Table 2). Additionally, a mRS ≥ 3 indicates moderate to severe disability or more severe life impairment. There were 3 patients (10.00%) with mRS ≥ 3 in the model group, and 10 patients (33.33%) in the control group (Table 2). Compare with the control group, the risk of aneurysm neck residual in the model group was reduced to 0.444 times, the risk of parent artery stenosis was reduced to 0.505 times, and the risk of mRS **≥** 3 was reduced to 0.553 times (Table 2). Overall, the surgical outcomes were superior in the model group compared to the control group.

**Table 2:** Comparison of surgical effects after 14-days surgery.

Group	Aneurysm neck remnant	Parent artery stenosis	mRS ≥ 3
Control group (*n* = 30)	6 (20.00%)	7 (23.33%)	10 (33.33%)
Model group (*n* = 30)	0 (0%)	1 (3.33%)	3 (10.00%)
*P*	**0.012**	**0.026**	**0.028**
OR	**0.444**	**0.505**	**0.553**
95% CI	**0.330–0.599**	**0.338–0.702**	**0.354–0.864**

Note: Bold values indicate statistical significance at *P* < 0.05.

### 3.2 Analysis of preoperative diagnostic accuracy of two groups

**Figure 2:**
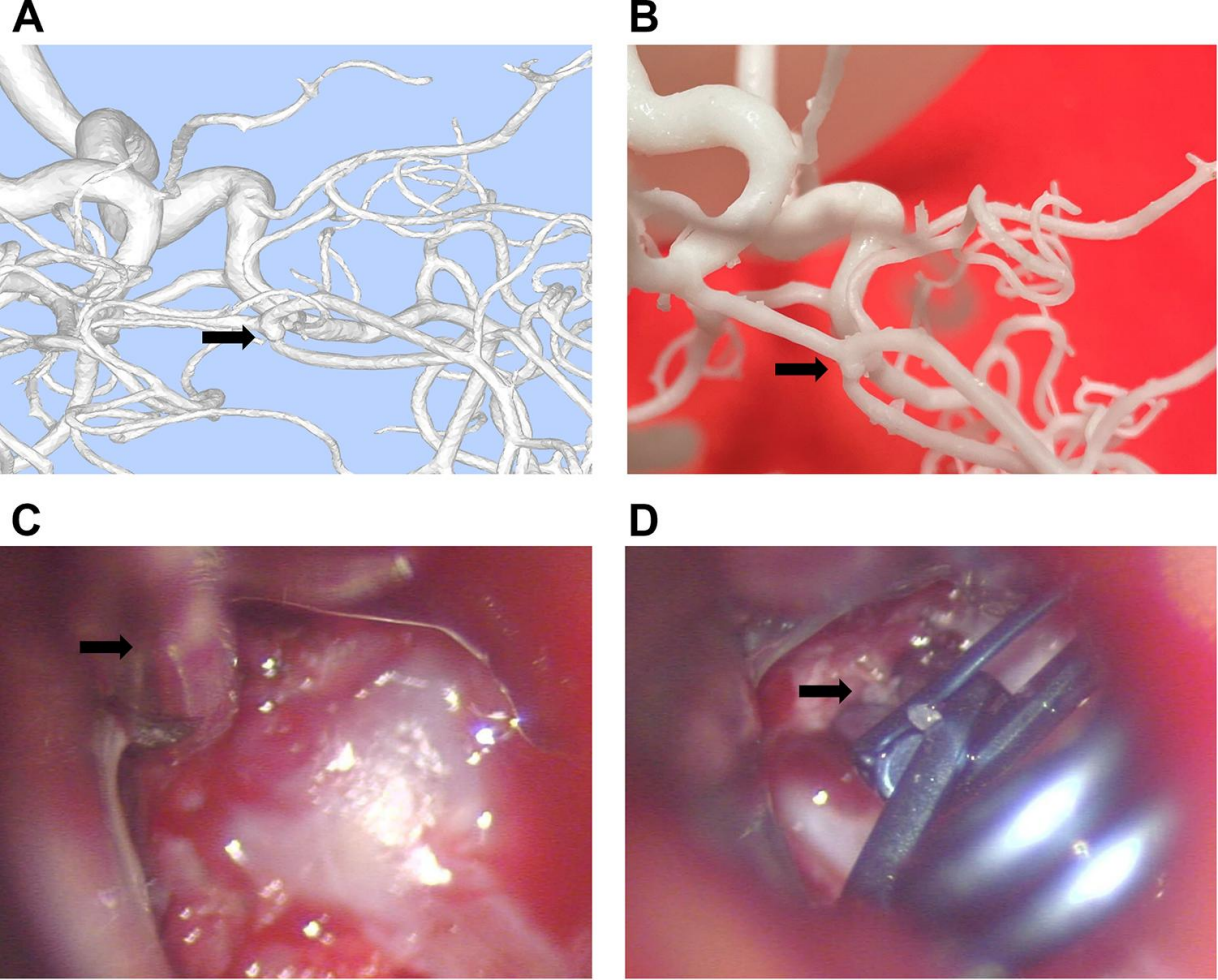
Consistency between the 3D printed model made from DSA imaging data and intraoperative observations. **(A)** 3D image reconstructed using dual-vessel DSA information via MIMICS software. **(B)** Aneurysm model created based on the reconstructed image. **(C)** Microscopic view during craniotomy shows the aneurysm closely matches the 3D printed model. **(D)** Image after aneurysm clipping during craniotomy.

For the control group, neurosurgeons assessed the morphology, size, angle, and relationships with surrounding tissues of the aneurysms based on DSA data. In the model group, DSA dual-vessel fusion reconstruction technology and 3D printing technology were used to create a 3D model that included the patient's aneurysm-supplying arteries, draining veins, and lesion information. This facilitated an analysis of the aneurysm's morphology, size, angle, and its relationships with surrounding tissues. It was observed that the 3D printed models, based on the patients' DSA imaging data, are basically consistent with the intraoperative observations (Figure 2). The model group had significantly fewer errors between preoperative diagnosis and intraoperative observations (Table 3). Among the 30 patients in the control group, there were 9 instances of diagnostic errors, including 2 cases of morphological errors, 1 case of neck measurement error, 1 case of orientation error, 2 cases of adjacency relationship error, and 3 cases of aneurysm clip selection error. In contrast, the model group reported only 1 orientation error and 1 morphological error. The risk of diagnostic error in the model group was 0.524 times that of the control group (Table 3). This indicates that the 3D printed model based on DSA can assist physicians in accurately assessing the actual condition of the aneurysm.

**Table 3:** Comparison of accuracy rates of preoperative diagnosis and aneurysm clip selection.

Group	Correct	Error	*P*	OR	95% CI
Control group (*n* = 30)	21 (70%)	9 (30%)	**0** **.** **02**	**0** **.** **524**	**0.342–0.803**
Model group (*n* = 30)	28 (93.33%)	2 (6.67%)			

Note: Bold values indicate statistical significance at *P* < 0.05.

### 3.3 Analysis of intraoperative conditions and hospital stay between the two groups

The average operation duration for the model group was 264.47 min, compared to 313.10 min for the control group. Patients in the model group had significantly shorter operation duration than those in the control group and Cohen's *d* was 0.865, which is a large effect (18) (Table 4). There was no significant difference in intraoperative blood loss and length of hospital stay between the model group and the control group. The potential of patient-specific 3D printed models to reduce intraoperative blood loss and hospital stay requires further investigation in a larger cohort (Table 4).

**Table 4:** Comparison of operation time and intraoperative blood loss between the two groups.

Group	Operation duration (min)	Surgical blood loss (mL)	Number of days in hospital (d)
Control group (*n* = 30)	313.10 ± 59.90	247.67 ± 125.55	25 (17.75, 30.25)
Model group (*n* = 30)	264.47 ± 52.27	205.50 ± 109.60	21 (18.00, 26.50)
*P*	**0.001**	0.171	0.394
Cohen's d/Z	**0.865**	0.358	−0.852
95% CI	**0.336–1.394**	−0.152–0.868	−2.000–6.000

Note: Bold values indicate statistical significance at *P* < 0.05.

### 3.4 Comparison of perioperative complications between the two groups

Postoperative complications are a key factor leading to poor patient outcomes. In the model group, one patient developed cerebral vasospasm and another developed epilepsy. In the control group, there were two cases of intraoperative aneurysm rupture, three strokes. Three patients experienced postoperative rebleeding, in addition, two patients developed epilepsy and three patients developed brain herniation (Table 5). When one patient experienced single or multiple complications, they were counted as one case. A total of 8 individuals (26.67%) experienced perioperative complications in the control group (Table 5). The risk of complications in the model group was 0.550 times that of the control group (Table 5). Overall, the total incidence of complications in the model group was lower than that in the control group.

**Table 5:** Incidence of perioperative complications among the patients.

Complications	Control group (*n* = 30)	Model group (*n* = 30)	*P*	OR	95% CI
Total number of people	8 (26.67%)	2 (6.67%)	**0** **.** **038**	**0** **.** **550**	**0.354–0.854**
Ruptured aneurysm	2 (6.67%)	0 (0%)			
Stroke	3 (10%)	0 (0%)			
Postoperative bleeding	3 (10%)	0 (0%)			
Cerebral vasospasm	5 (16.67%)	1 (3.33%)			
Epilepsy	2 (6.67%)	1 (3.33%)			
Cerebral herniation	3 (10%)	0 (0%)			

Note: Bold values indicate statistical significance at *P* < 0.05.

## 4 Discussion

To date, there has been no report on whether the preparation of aneurysm clipping surgery guided by 3D-printed models for ACoA aneurysms can improve patient outcomes. In this prospective cohort study, we found that 3D printing technology based on the fusion of dual-vessel DSA images can create patient-specific models with a high degree of fidelity. The models' display of aneurysm shape, size, and adjacent relationships were basically consistent with intraoperative observations, providing an effective surgical practice simulation tool for resident neurosurgeons. This can have a positive impact on improving surgical outcomes and reducing trauma.

Studies have shown that 3D printing technology offers new possibilities for customized individual medical solutions (19, 20). For example, creating patient-specific 3D printed models based on CT for proximal humeral fractures can improve surgical outcomes and reduce costs (21). Similarly, models of cardiac and pericardiac tumors created from CT and CTA provide more information compared to traditional imaging, effectively aiding in preoperative planning (22). 3D printed models for patients with visceral aneurysms based on CTA facilitate a more convenient assessment of the aneurysm sac's position, morphology, and geometry, encouraging surgeons to perform endovascular procedures with favorable results (23). These studies indicate that patient-specific 3D printed models are beneficial tools for improving individualized treatment and reducing surgical risks.

3D printing technology has unparalleled advantages for the education and training of neurosurgical residents, as well as for patient diagnosis and treatment (24). For example, the use of 3D-printed models to assist with the precise shaping of microcatheters and preoperative rehearsals can make the coil embolization of intracranial aneurysms simpler and safer (9, 10). Recent studies have found that preoperative planning with 3D-printed models based on CTA can significantly reduce average surgery time for aneurysm clipping at the anterior communicating artery, posterior communicating artery, middle cerebral artery, and internal carotid artery (7). Compared with previous studies, it was found that 3D printed models used in preoperative communication or surgical training for aneurysms mostly used specific models, while the use of patient-specific personalized models for preoperative simulation or surgical planning was mainly targeted at patients with unruptured aneurysms (Table 6). This study utilized DSA to create 3D printed models that provide high-resolution vascular information. The printing precision is sufficient to fabricate perforator arteries with a minimum diameter of 0.5 mm. Furthermore, the model also replicates multiple blood supply arteries of the ACoA aneurysm. This offers a more accurate representation of the patient's anatomy. The time required to produce the 3D printed model is also crucial for its clinical applicability. In this study, patient-specific models can be completed within 2–8 h and delivered to the attending physician. This represents a timelier model production compared to previous studies (7, 25). This means that the model applicable even for patients with ruptured aneurysms require urgent surgery.

**Table 6:** Application of 3D printing models in neurosurgery for aneurysms.

References	Experiment type	Template	Application scope	Main results
Joseph FJ et al., (26)	Quasi-experimental research	CT, MRI, and DSA	A complex 3D printed aneurysm model for patient education in aneurysm clipping surgery	Improve communication and enhance patient understanding of the pathology and its treatment
Wang JL et al., (27)	Quasi-experimental research	DSA	Specific 3D printed models for aneurysm treatment and residency training	Assist surgery and improve training results
Mashiko T et al., (28)				
Li SS et al., (29)	Randomized controlled trials	CTA/MRI	A database of 3D printed models of cerebral aneurysms with various morphologies for training in microcatheter angioplasty for cerebral aneurysms	Improve training effectiveness
Wang S et al., (30)	Randomized controlled trials	DSA/CTA	Unruptured aneurysm patient brain anatomy model or vascular anatomy model for preoperative simulation of clipping and endovascular treatment	Shorten operation time, reduce postoperative complications, and reduce postoperative neurological dysfunction
Woo SB et al., (31)				
Marciuc EA et al., (13)	Randomized controlled trials	CTA	Patient-specific 3D-printed models guide endovascular treatment of intracranial aneurysms	Help select endovascular treatment techniques and optimize treatment plans
Song X et al., (10)				

The reduction in intraoperative vasospasm observed in our model group (3.33% vs. 16.67% in the control group) is particularly noteworthy. This finding aligns with recent literature suggesting that excessive manipulation of vascular structures during aneurysm surgery is a significant contributor to intraoperative vasospasm. Studies have reported that approximately 13% of patients with unruptured aneurysms experience vasospasm after clipping, which is related to the vasoconstrictive effect caused by the surgical procedure (32). The precise preoperative understanding of aneurysm anatomy afforded by our 3D models likely facilitated more direct approach trajectories, minimized unnecessary vessel manipulation, and reduced the risk of vasospasm. This benefit is particularly important for ACoA aneurysms, where the complex perforator anatomy often necessitates extensive dissection when the approach is not optimally planned.

Utilizing 3D-printed models for preoperative simulation may improve postoperative outcomes for patients from the following aspects. First, neurosurgeons can use 3D-printed models to explain the specifics of the aneurysm and the surgical plan to patients and their families, which can facilitate a better understanding of the impending operation, increase patient cooperation, and reduce anxiety (33). Second, prior to surgery, the neurosurgeons in the model group can diagnose the morphology, size, angle, and relationship with surrounding structures of the aneurysm with precision based on the patient's 3D-printed model, thereby refining the surgical approach. This includes avoiding important arteries during the separation of the subarachnoid space, orienting the aneurysm, and aiming to expose the parent artery and the neck of the aneurysm as much as possible. Previous studies have indicated that employing an approach that eases observation, and manipulation can effectively decrease the risk of aneurysm rupture and improve the clamping efficiency on the aneurysm neck (34). Third, in the model group, the physician will perform clipping simulations based on the patient's model, focusing not only on the size of the aneurysm clip but also analyzing the placement and angle of the clip, which could help avoid unnecessary risks caused by intraoperative adjustments (35). Research shows that residual neck post-clipping is a critical risk factor for secondary treatment (36), and repeated clip placements may lead to adverse outcomes such as clip slippage or aneurysm rupture (37). Therefore, determining the clip size, placement position, and angle in advance is crucial for avoiding risks such as neck remnants and aneurysm rupture. Lastly, in some complex aneurysm surgeries, 3D-printed models can even serve as intraoperative reference tools, assisting surgeons in navigating through complex anatomical structures, thus reducing operative time and intraoperative blood loss. This, by minimizing the exposure time of brain tissue, can lead to faster patient recovery and a decreased risk of infection (38). In summary, we believe that 3D-printed models based on the fusion of dual-vessel DSA provide effective technical support in ACoA aneurysm clipping surgeries. As imaging technologies and model manufacturing techniques continue to advance, these models may demonstrate even greater potential and value and are worthy of further promotion.

## 5 Limitations

We acknowledge several limitations of this study. First, this study is the small sample size. The fact that patients were not subdivided according to the rupture condition limited our ability to make meaningful statistical comparisons and draw definitive conclusions in particular population. The non-randomized design and single-center nature limit the generalizability of our findings. The grouping method of whether patients are willing to participate in 3D printing model construction as a preoperative auxiliary diagnostic tool may bring the risk of selection bias and insufficient sample size. Secondly, this study did not include long-term follow-up, which limits the exploration of the impacts on long-term complications or aneurysm recurrence. Third, all surgeries were performed by a single experienced neurosurgeon, which may not reflect the potential utility of 3D models for surgeons at different experience levels. Finally, the time constraints for model production remain a significant issue; in emergency situations, it may only be feasible to print the localized ACoA aneurysm and its feeding arteries, potentially leading to the loss of important anatomical details. This study preliminarily reveals the potential application value of 3D printing technology in the microsurgical clipping of ACoA aneurysm. With the widespread clinical application of 3D-printed models, there is hope for further investigation of their effectiveness and safety in larger prospective cohorts.

## 6 Conclusion

The specific benefits observed in our study include a trend toward fewer perioperative complications and higher rates of complete aneurysm occlusion in the group using 3D-printed models. This prospective cohort study demonstrates that DSA-derived 3D printed models can enhance the understanding of ACoA aneurysm anatomy and potentially optimize surgical planning. Although this technique shows potential in reducing perioperative complications and promoting patient recovery, its exact efficacy still needs to be verified by larger randomized controlled trials.

Future research should prioritize multicenter randomized controlled trials with larger sample sizes and longer follow-up periods to validate our findings. Additionally, cost-effectiveness analyses and investigations into the utility of this technology for less experienced surgeons would provide valuable insights for broader adoption. It remains to be explored whether obtaining a comprehensive skull/brain/vasculature model within a limited timeframe can provide more effective surgical guidance. Research in these directions will help advance the role of 3D-printed models in precision neurosurgery.

## Data Availability

The original contributions presented in the study are included in the article/Supplementary Material, further inquiries can be directed to the corresponding author.
